# Quantifying the economic cost of antibiotic resistance and the impact of related interventions: rapid methodological review, conceptual framework and recommendations for future studies

**DOI:** 10.1186/s12916-020-1507-2

**Published:** 2020-03-06

**Authors:** Mark Jit, Dorothy Hui Lin Ng, Nantasit Luangasanatip, Frank Sandmann, Katherine E. Atkins, Julie V. Robotham, Koen B. Pouwels

**Affiliations:** 1 The National Institute for Health Research (NIHR) Health Protection Research Unit in Immunisation, London School of Hygiene & Tropical Medicine, Keppel Street, London, WC1E 7HT UK; 2 Modelling and Economics Unit, National Infections Service, Public Health England, London, UK; 3 School of Public Health, University of Hong Kong, Hong Kong, SAR China; 4 Department of Infectious Diseases, Singapore General Hospital, Singapore, Singapore; 5 Mahidol-Oxford Tropical Medicine Research Unit, Faculty of Tropical Medicine, Mahidol University, Bangkok, Thailand; 6 Centre for Global Health Research, The Usher Institute for Population Health Science and Informatics, The University of Edinburgh, Edinburgh, UK; 7 The National Institute for Health Research (NIHR) Health Protection Research Unit in Healthcare Associated Infections and Antimicrobial Resistance, University of Oxford, Oxford, UK; 8 The National Institute for Health Research (NIHR) Health Protection Research Unit in Healthcare Associated Infections and Antimicrobial Resistance, Imperial College London, London, UK; 9 Health Economics Research Centre, Nuffield Department of Population Health, University of Oxford, Oxford, UK

**Keywords:** Antimicrobial resistance, Antibiotics, Economic costs, Economic evaluation

## Abstract

**Background:**

Antibiotic resistance (ABR) poses a major threat to health and economic wellbeing worldwide. Reducing ABR will require government interventions to incentivise antibiotic development, prudent antibiotic use, infection control and deployment of partial substitutes such as rapid diagnostics and vaccines. The scale of such interventions needs to be calibrated to accurate and comprehensive estimates of the economic cost of ABR.

**Methods:**

A conceptual framework for estimating costs attributable to ABR was developed based on previous literature highlighting methodological shortcomings in the field and additional deductive epidemiological and economic reasoning. The framework was supplemented by a rapid methodological review.

**Results:**

The review identified 110 articles quantifying ABR costs. Most were based in high-income countries only (91/110), set in hospitals (95/110), used a healthcare provider or payer perspective (97/110), and used matched cohort approaches to compare costs of patients with antibiotic-resistant infections and antibiotic-susceptible infections (or no infection) (87/110). Better use of methods to correct biases and confounding when making this comparison is needed. Findings also need to be extended beyond their limitations in (1) time (projecting present costs into the future), (2) perspective (from the healthcare sector to entire societies and economies), (3) scope (from individuals to communities and ecosystems), and (4) space (from single sites to countries and the world). Analyses of the impact of interventions need to be extended to examine the impact of the intervention on ABR, rather than considering ABR as an exogeneous factor.

**Conclusions:**

Quantifying the economic cost of resistance will require greater rigour and innovation in the use of existing methods to design studies that accurately collect relevant outcomes and further research into new techniques for capturing broader economic outcomes.

## Background

For several decades now, it has been known that antibiotic resistance (ABR) among human pathogens is detrimental to health and economic wellbeing [1–3]. International recognition of its threat to modern medicine and society has increased in recent years [4–7]. Several national and international working groups have proposed actions to mitigate its further development, with recommendations including novel funding structures for new antibiotic research and development, development and use of technologies like diagnostics and vaccines that may reduce the need for antibiotics, education to enhance antibiotic stewardship as well as better infection control practices such as ensuring universal access to sanitation, hygiene and safe water [4–7].

Many of the societal conditions that have increased the threat of ABR, such as a lack of investment in antibiotic development and antibiotic overuse, stem from market failure, i.e. the private interests of individuals conflicting with the public interests of society. When used appropriately to treat bacterial infections, antibiotics generate private (individual) benefits by expediting infection clearance and recovery from associated illness, whilst also generating public benefits by reducing the spread of infection across the rest of the population (positive externalities). However, these public benefits are usually outweighed by the costs that fall on others who do not use them (negative externalities) — their use can select for bacteria with more resistance, hence eroding the effectiveness of future antibiotic use. In other words, using antibiotics consumes the global stock of antibiotic effectiveness, making antibiotics less beneficial to everyone who uses them. Additionally, antibiotics confer no benefits against viral infections (and indeed bacterial infections that are resistant to that antibiotic). Since there is usually uncertainty about the aetiology and susceptibility of an infection, patients may demand antibiotic prescriptions and their prescribers are often incentivised to respond to this demand whether it be for a bacterial or viral infection.

Because self-interested antibiotic consumers have little motivation to conserve the global stock of antibiotic effectiveness, most of the recommended interventions to combat ABR are unlikely to succeed without government intervention at the national and international levels. Indeed, even when prescribers act as custodians for antibiotic consumption, they are predicted to prescribe antibiotics to patients even when they only have a small chance of benefit [8].

To correct these market failures and align individual and societal interests, governments (and other healthcare funders such as donors and private insurers) may incentivise prudent antibiotic use through taxes, subsidies and regulations, and fund research in antibiotics and other technologies [9]. However, the scale of these interventions needs to be calibrated to the size of the negative externality, such that it reflects the social value of prolonging antibiotic effectiveness. Striking this balance requires an accurate understanding of the economic cost of ABR [10].

Several reviews have examined the cost of ABR. In particular, five reviews covering the periods 1995–2009 [11], 1987–2000 [12], 2000–2012 [13], 2013–2015 [14] and 2000–2016 [15] found 36, 21, 24, 11 and 8 studies, respectively, assessing the cost of ABR and/or the cost-effectiveness of related interventions. The reviews highlighted methodological shortcomings and biases in the literature as well as challenges in extrapolating study findings to a societal scale and to the long term. In particular, the heterogeneity in methods and quantitative results across different studies complicates meaningful comparisons [11, 14, 16, 17]; this heterogeneity is present even when restricting comparisons to studies of a particular organism within the hospital setting [18].

Despite these issues in interpreting and quantifying the costs of ABR, none of the reviews provided detailed proposals of how the evidence gaps and study heterogeneities could be addressed in future studies. Indeed, a lack of certainty on the current and potential future economic burden of ABR, especially in low- and middle-income countries, has been recognised to have hindered international action [4].

Several estimates have recently been made of the global ABR burden to present the case for investing in measures to combat ABR [7, 19, 20]. These global estimates were performed largely independently of the hospital-based literature, relying instead on international surveillance databases that have been noted to suffer from representativeness issues, confounding and biasedness around attributable mortality and ABR prevalence as well as contestable costing assumptions [21]. Furthermore, quantifying the economic burden of ABR on its own is of little value for decision-making unless it can inform estimates of the impact that different interventions can have on this burden.

To overcome these evidence gaps, we present a conceptual framework that can act as a roadmap for future studies to describe how they can reduce bias and be extended in time, perspective, scope and space. We use this framework to develop comprehensive recommendations for the design of future studies that will allow policy decisions based on a clearer and more consistent methodology as well as on more comprehensive evidence. Our framework is supplemented by a rapid methodological review of studies that consolidates articles in previous reviews on ABR costs and the impact of ABR-related interventions.

## Methods

### Conceptual framework

Previous reviews [11–15] have highlighted methodological shortcomings in the ABR cost literature, including heterogeneity in methodological choices [11, 14, 15], biases in study design and analysis [11, 14], lack of evidence outside high-income [12, 15] and hospital [12, 13] settings, and failure to consider the future consequences of ABR [13]. We used these critiques to develop a conceptual framework of how current studies could be strengthened and extended to address these shortcomings. This was supplemented by additional deductive epidemiological and economic reasoning. The deductive reasoning was guided by the literature on causal inference [14, 22–26], perspective and scope of economic impact [27], and opportunity costs [28]. We then conducted a rapid methodological review to (1) extract broad features of ABR cost studies relevant to the methods they use, (2) survey the methods used to cost ABR and (3) examine the extent to which these methods addressed methodological shortcomings. A narrative synthesis of these methods and their limitations was developed and then used to further modify our conceptual framework.

### Rapid methodological review

To inform the conceptual framework, we conducted a rapid methodological review of published and grey literature. For published literature, we searched PubMed and Ovid MEDLINE for all studies up to November 4th, 2019 (see Additional file 1 for search terms). For grey literature, the websites for the World Bank (www.worldbank.org), the European Centre for Disease Prevention and Control (www.ecdc.europa.eu), and the Centre for Infectious Disease Research and Policy (www.cidrap.umn.edu) were examined. We also reviewed the reference lists of five related reviews [11–15]. Studies were not screened in duplicate.

We included all English-language articles (including conference abstracts in peer-reviewed journals) that quantified the cost of ABR from any economic perspective, including papers that quantified these costs as part of an economic evaluation of an intervention to reduce ABR. Both primary data and secondary data analyses were included as long as real costs were an outcome. We included studies that calculated the incremental costs of treating patients with resistant infections relative to either treating patients with a susceptible infection or no infection at all.

Exclusion criteria were (1) studies about ABR in *Mycobacterium tuberculosis* alone (due to the large volume of this literature using techniques relevant to tuberculosis alone) or non-bacterial pathogens only; (2) studies about bacteria in non-human species alone; (3) reviews, commentaries or theoretical papers that did not estimate a cost associated with ABR that is applicable in a real-world setting; (4) studies that calculated the cost of an intervention (e.g. use of an antibiotic) in the presence or absence of ABR rather than the actual cost of ABR; (5) studies that calculated the cost in terms of total antibiotic use and/or prescription expenditure alone rather the cost associated with a resistant infection; and (6) studies that were not accessible to the authors.

Articles that discussed approaches to quantify costs but did not present numerical results were also examined to inform the wider discussion.

The data extracted from each study is shown in Additional file 2. Descriptive summary statistics were calculated on publication date, country, healthcare setting, economic perspective, bacterial species and study design. Full text papers were read for details on study design and recommendations. A reporting checklist of the review methodology is shown in Additional file 3.

## Results of rapid methodological review

### Descriptive results

**Fig. 1:**
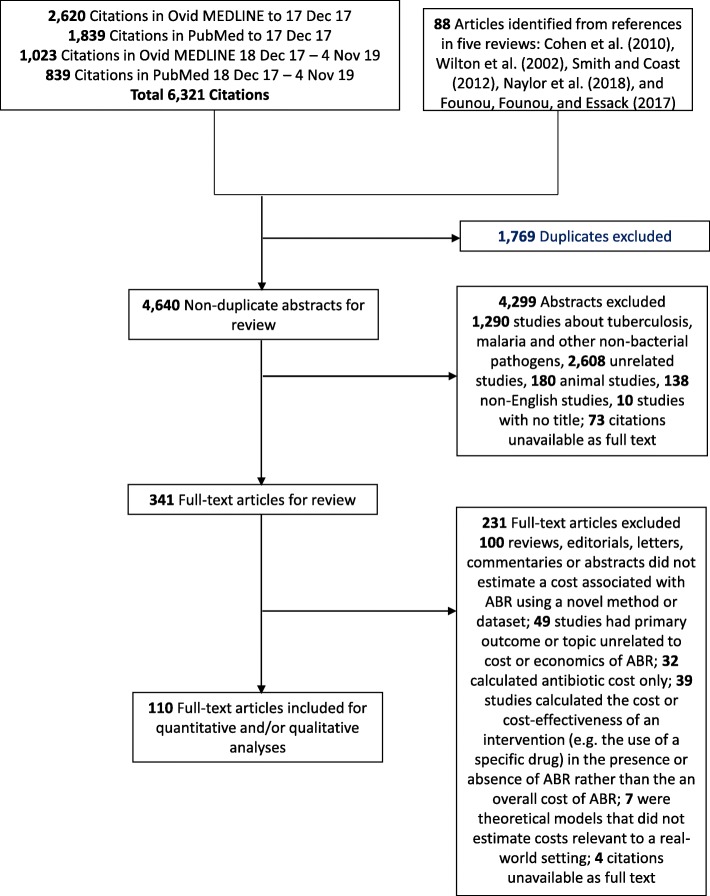
PRISMA flow diagram showing the review process

We found 6347 articles from combining the database search and references from previous reviews. Of these, 110 articles were included following abstract and full-text review (Fig. 1; details in Additional file 2), including 43 articles that had not been included in any previous review.

The articles were published during 1998–2019, with half (55/110) being published since 2011. Most (91/110) presented results for high-income countries only, with almost half (48/110) from the United States. Only 12 were from low- and middle-income countries (Brazil, China, Columbia, Ethiopia, India, Senegal, Thailand and Turkey), while 7 included multi-country results. Most (95/110) were set in hospitals (8 in intensive care units), with the remainder being from community care (either acute or long-term) or mixed settings. Most (97/110) used a healthcare provider or payer perspective, with the remainder using either a societal perspective (8/110) or multiple perspectives (5/110). The most common (87/110) method for costing was using pairwise matching or regression on a patient cohort, while the remaining studies used a cohort exclusively of patients with resistant infections (1/110), ecological analysis or meta-regression of multiple studies (4/110), or mathematical modelling (18/110) to estimate costs.

Around half (52/110) examined Gram-positive bacteria alone, with *Staphylococcus aureus*, *Enterococci* and *Streptococcus pneumoniae* being the most common. Fewer (29/110) examined Gram-negative bacteria alone, with Enterobacteriaceae, *Pseudomonas aeruginosa* and *Acinetobacter baumannii* being the most common. The remainder looked at mixed groups of patients with both Gram-positive and Gram-negative bacteria.

### Hospital-based studies

Similar to previous reviews [12–15, 22], we found that most studies focused solely on treatment costs incurred by hospital patients with antibiotic-resistant infections, using one of two designs:

- A matched cohort design, where patients with antibiotic-resistant and antibiotic-susceptible infections (or no infection at all) are matched and their cost outcomes compared. Matching is based on patient characteristics and differs widely across studies; some have matched only on patient age and gender, while others have used diagnostic codes, severity-of-illness scores or length of stay from admission to infection onset. However, such studies rarely explore whether the matching characteristics are good indicators of the risk of acquiring an antibiotic-resistant infection [22].
- Regression on a patient cohort to link the presence of an antibiotic-resistant infection with cost-related explanatory variables. In both cases, the outcome variable is treatment costs (calculated using hospital charges and/or standard reimbursement tariffs) or an intermediate outcome such as length of stay, which is then used to calculate costs. The latter approach may underestimate costs by ignoring other cost elements that may be greater for antibiotic-resistant infections such as testing and prescription costs, management of complications, and isolation ward and intensive care stays [18].

Many studies suffer from methodological shortcomings that may either underestimate or overestimate of the actual cost of ABR, as has been pointed out previously [14, 17, 23]. These shortcomings include (1) unmeasured baseline confounding (not adjusting for differences in the characteristics of patients with antibiotic-resistant, antibiotic-susceptible or no infection that affect their clinical outcomes or treatment costs), (2) time-dependent confounding (patients with antibiotic-resistant, antibiotic-susceptible or no infections have different changes to their characteristics such as health between the time of admission and acquiring an infection) [24, 25], (3) time-dependent bias (patients with longer stays being more likely to acquire antibiotic-resistant infections) [25], and (4) model misspecification (use of inappropriate models to relate variables to outcomes, such as the use of Cox regression models even though the proportional hazards assumption is rarely valid for cost outcomes) [26].

The literature in this area has shown methodological improvements over time, with greater use of techniques that can correct for time-dependent biases, such as survival models incorporating infection as a time-dependent predictor and multi-state models [29]. However, few studies adjust for potential time-varying confounding when focusing on hospital-acquired cases, using g-methods such as marginal structural models with inverse probability weighting [25] or nested g-formulae [24]. In contrast to standard regression methods and multistate models, g-methods can provide unbiased estimates of an exposure if there is time-varying confounding that is also affected by the exposure, provided that confounding is accurately measured.

The use of regression and propensity score matching rather than paired cohorts has also increased, which should reduce residual confounding since paired cohorts can only control for a limited selection of variables. Instrumental variable approaches may offer a more powerful approach to correct for observable and unobservable confounders [22]. However, none of the reviewed studies used suitable instruments. It may be possible to use rapid policy shifts, such as changes to hospital cleaning regimens, as instruments, but these shifts would need to be sufficiently large and may be confounded by other time trends.

## Conceptual framework

**Fig. 2:**
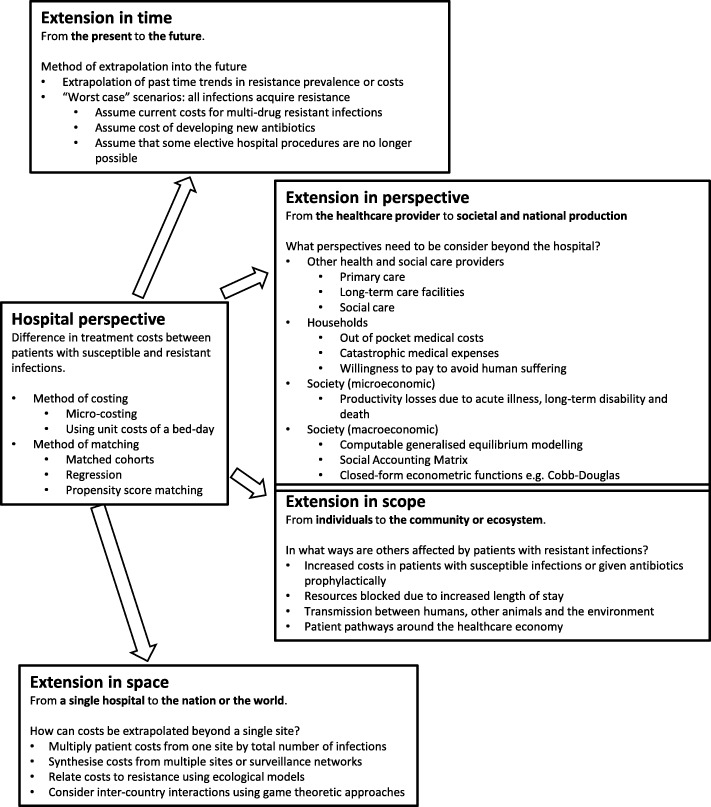
Conceptual framework outlining options discussed in the text to extend the scope of studies estimating the costs of antibiotic resistance (see text for references)

A major limitation of hospital-based studies is that, on their own, they are insufficient to fully capture ABR economic burden on a national or global level or to assess the impact of interventions against the development of ABR. The conceptual framework proposed describes how they can be extended in time, perspective, scope and space (Fig. 2).

### Extension in time: from the present to the long term

Based on present trends, the prevalence of ABR, and especially multi-drug resistance, is expected to rise [30–32]. However, most estimates of current ABR economic burden do not project estimates into the future, and hence fail to adequately capture the value of interventions that may avert catastrophic future scenarios.

A few studies have taken scenario-based approaches by (1) assuming that future ABR prevalence and/or disease prevalence will increase by arbitrary amounts simply to explore what the consequences of such scenarios would be [7, 19, 20], (2) assuming that all currently susceptible bacterial strains will acquire ABR in the future [33], or (3) linearly projecting past data on disease incidence and ABR prevalence [31, 32].

Even the most extreme scenarios may not represent the worst case, because these projections do not account for new resistances that bacteria may acquire, including resistance to current last-resort antibiotics. If this happens, then the cost of developing new antibiotics or antibiotic classes needs to be incorporated. Otherwise, some analysts have speculated that hospital procedures, such as organ transplantation and cancer chemotherapy, will become risky to perform because of untreatable infections. Approaches to costing such scenarios have been discussed [16, 19, 34], but no study has presented quantitative results of such calculations.

### Extension in perspective: from the healthcare payer to society

Most hospital-based studies only consider ABR costs from the perspective of the healthcare provider (e.g. the hospital providing treatment). Other perspectives are arguably relevant to decision-makers considering whether to fund investments with national and global implications such as developing a new antibiotic.

One such perspective is that of the patient’s household. If ABR results in longer hospital stays and more complex procedures, then this may increase household out-of-pocket costs such as co-payments for treatment, transport costs, caregiver accommodation costs and childcare costs. Patients and caregivers may miss work and lose income. These costs may be especially large (in comparison to existing income or wealth) for poorer patients, especially those in countries without universal health coverage [35]; none of the reviewed papers considered these distributional perspectives. Such distributional concerns should also be considered in the use of interventions to correct for market failures in antibiotic use. For instance, taxing antibiotics may particularly burden the poor since the demand for antibiotics is relatively income inelastic [36]; therefore, compensatory measures may be appropriate such as funding healthcare services and improvements in infection prevention strategies that will reduce the overall need for antibiotics [10].

A further dimension of household economic burden is the value of avoiding pain, suffering and worry to patients and caregivers, including the value of avoiding premature mortality. In economic evaluations, this is usually done by either (1) monetising this value by eliciting people’s willingness to pay to avoid morbidity and mortality or (2) valuing it using a separate metric such as quality- or disability-adjusted life years. As an example, Phelps [37] costed each avoided death as having a value of US$1 m (2 m in 2017 I$). Capturing household and psychosocial aspects of ABR burden is especially important in low- and middle-income settings where treatment costs and hospitalisation rates may be lower (due to low labour costs and poor healthcare access, respectively), but the human cost of ABR remains high.

The societal perspective is the broadest and includes the effect that morbidity and mortality have on economic production. Only 8/110 of the reviewed studies included explicitly mentioned incorporating productivity costs; those that did, concluded that productivity costs were greater than direct medical costs. Most (6/110) used the human capital method, whereby any time lost from work as a result of death, sickness or caregiving is converted into production costs by multiplying either by the average wage or by the national GDP per capita; none subtracted future medical or non-medical consumption as recommended by some economists [38]. Two studies argue that the human capital method underestimates the impact of ABR to society because it ignores the broader macroeconomic impact of reduced labour supply, increased healthcare demand and reduced consumption due to sickness, disability, premature mortality and fear of infection. Increased rates of ABR are likely to have a detrimental effect on unemployment, inflation, total factor productivity and, ultimately, national income [9]. There are a number of ways through which studies have tried to incorporate these macroeconomic effects — capturing labour and capital stocks with the Cobb–Douglas function [19], modelling intersectoral flows with Social Accounting Matrix methods [19, 20] and dynamically capturing economic shocks with Computable General Equilibrium methods [9, 39].

Additionally, when taking the societal perspective, disease not resulting in hospitalisation (i.e. treated in the community, in primary care or in outpatient clinics) becomes important because this may still result in productivity losses even if direct healthcare costs are low.

### Extension in scope: from the individual patient to the community

Most studies have considered only the difference in costs of treating patients with antibiotic-susceptible and antibiotic-resistant infections; however, ABR can also increase costs for patients without antibiotic-resistant infections. For instance, patients with infections for which ABR is widespread are routinely given costly ABR tests and/or empirically prescribed antibiotics that are costlier and have worse side effects than first-line antibiotics. One study found that ABR contributed to a 22% increase in per prescription antibiotic spending on otitis media [40].

Some antibiotics are given prophylactically to patients particularly susceptible to infections such as pregnant women [41] and cancer or surgical patients [34, 42]; loss of antibiotic effectiveness will decrease prophylaxis effectiveness and worsen patient outcomes [34]. These effects can be translated into costs [16], although no paper we reviewed has done this explicitly.

By considering only the incremental cost of treating antibiotic-resistant infections compared to antibiotic-susceptible infections, most studies imply that, even if ABR was eliminated, patients would still be infected by a susceptible infection. However, bacterial transmission is often driven by the ineffectiveness or slow effectiveness of antibiotic treatment, causing antibiotic-susceptible strains to spread more slowly; thus, reducing ABR may also reduce overall infection incidence.

Further spill-overs occur from the opportunity costs associated with antibiotic resistance to others in the community who may be unable to access timely healthcare when resources, such as hospital beds, are blocked by patients with longer stays due to resistant infections. These opportunity costs can be expressed in terms of monetary time costs or in terms of impact on hospital revenues [43] or the health of other patients [28].

Finally, resistant infections arise and are transmitted within and between humans, non-human animals and the environment. Patients with resistant infections can move between different healthcare sectors such as hospitals, intermediate/long-term care and the community. These effects can influence the impact of interventions; the methods to capture them are described in more detail in the section on “The impact of interventions” below.

### Extension in space: from one hospital to national/global estimates

Antibiotic-resistant organisms cross boundaries between host species (humans and other animals), hospitals and countries. Single hospital studies may be useful to estimate the cost of a localised outbreak, but the impact of policy instruments usually needs to be considered at the national or even global level. Game-theoretic analyses suggest that optimal antibiotic allocation requires cooperation between countries rather than allowing individual patients or even countries to act in their own self-interest [44].

Only 18/110 of the reviewed studies (of which only 7 were published before 2018) and a further 4 studies found in the grey literature extrapolated findings from study sites to countries or multi-country groupings (Table 1). The most common approach is to multiply incremental costs in study sites by the total number of antibiotic-resistant cases across the entire geography. The shortcoming of this approach is that data from a single hospital or even several hospitals often lack external validity [32]. Quantitative comparisons of the literature have found substantial differences in costs between studies [11, 17, 22]. Another approach is to synthesise information from multiple studies as was done in cross-European analyses [6]. Since studies are heterogeneous, in the long term, multi-centre studies with a common methodology may improve both statistical power and validity beyond study settings [22].

**Table 1:** Estimates of national, multinational and global costs of antibiotic resistance. Costs were inflated using local GDP deflators and converted to 2018 international dollars (I$) using purchasing power parities, both from data published by the World Bank and OECD

Study	Geography	Pathogens	Costs in original currency	Costs in 2018 I$	Costs considered
Studies in the grey literature					
ECDC and EMEA [6]	EU, Iceland, Norway	*S. aureus*, *Enterococcus spp.*, *S. pneumoniae*, *E. coli*, *Klebsiella* spp., *P. aeruginosa*	1.5 bn/year EUR (2007)	2.1 bn/year	Increased treatment costs, reduced productivity and labour supply due to morbidity and premature mortality
KPMG [19]	EU, Iceland, Norway Global	*E. coli*, *K. pneumoniae*, *S. aureus*, HIV, TB, malaria	1.6 bn/year EUR (2012) 1.66–6.08% of global GDP in 2050	2.3 bn/year	Increased treatment costs, reduced productivity and labour supply due to morbidity and premature mortality
RAND Europe [20]	Global	*E. coli*, *K. pneumoniae*, *S. aureus*, HIV, TB, malaria	0.5–6.0 tn USD (2011) per year in 40 years (0.14–1.9% of global GDP)	0.6–6.8 tn per year in 40 years	Reduced labour supply and productivity due to increased morbidity, mortality and caregiving Reduced inter-sectoral transactions and trade
World Bank [7]	Global	Any	1.0–3.4 tn USD (2017) per year in 2030 (1.1–3.8% of global GDP)	1.0–3.5 tn I$ per year in 2030	Reduced labour supply due to premature mortality
Studies in the rapid review of published literature					
Chen et al. [45]	Ethiopia	*S. pneumoniae*	15.8 m/year USD (2017)	16.2 m/year	Increased treatment costs and productivity losses due to morbidity and premature mortality
Chesson et al. [46]	USA	*N. gonorrhoeae*	378 m/year USD (2016)	395 m/year	Increased treatment costs
de Kraker et al. [32]	31 European countries	Bloodstream infections caused by MRSA and G3CREC	62 m/year EUR (2007)	87 m/year	Increased length of hospital stay
Elbasha [47]	USA	Any	0.4–19 bn/year USD (1996)	0.6–29 bn/year	“Deadweight loss”: reduced antibiotic effectiveness leading to poorer treatment outcomes due to overprescribing antibiotics
Johnston et al. [48]	United States	Multi-drug resistant organism	2.39–3.38 bn/year USD (2017)	2.45–3.46 bn/year	Increased treatment costs
Lee et al. [49]	USA	Community-associated MRSA	Healthcare: 478 m/year USD (2011) Society: 2.2 bn/year USD (2011)	Healthcare: 539 m/year Society: 2.5 bn/year	Increased treatment costs and productivity loss due to morbidity and premature mortality
Michaelidis et al. [50]	USA	Any	4.4 bn/year USD (2013)	4.8 bn/year	Cost of antibiotic use and stewardship
Naylor et al. [29]	England	*E. coli*	Third-generation cephalosporin: 366,600/year GBP (2012) Piperacillin/tazobactam: 275,4000/year GBP (2012)	Third-generation cephalosporin: 578,000/year Piperacillin/tazobactam: 434,000	Increased treatment costs
Phelps [37]	USA	Any	0.15–3 bn/year USD (1984)	0.3–6.3 bn/year	Treatment costs, mortality
Phodha et al. [51]	Thailand	Nosocomial infections due to five bacterial species	Healthcare: 2.3 bn/year USD (2012) Society: 4.2 bn/year USD (2012)	Healthcare: 2.5 bn/year Society: 4.6 bn/year	Increased treatment costs Increased societal costs (components not reported)
Shrestha et al. [52]	USA and Thailand	Any	USA: 2.9 bn/year USD (2016) Thailand: 0.5 bn/year USD (2016)	USA: 3.0 bn/year Thailand:0.5 bn/year	Increased treatment costs and productivity loss due to morbidity and premature mortality
Smith et al. [39]	UK	MRSA	0.4–1.6% of national GDP, equivalent to 3–11 bn GBP (1995)	6.5–24.0 bn	Reduced labour supply and productivity, leading to less capital investment and lowered productivity
Thorpe et al. [53]	USA	Any	2.2 bn/year USD (2016)	2.3 bn/year I$	Increased treatment costs due to morbidity
Tillekeratne et al. [54]	Sri Lanka	Any	229 m/year USD (2017)	235 m/year	Not specified – costs extrapolated from US and Thai studies
Touat et al. [55]	France	Gram-negative bacteria	287 m/year EUR (2015)	397 m/year	Increased treatment costs
US Congress, Office of Technology Assessment [56]	USA	Nosocomial infections due to six bacterial species	1.3 bn/year USD (1992)	2.1 bn/year	Hospital treatment costs
Zhen et al. [57]	China	Intra-abdominal bacterial infections	Healthcare: 37 bn/year CNY (2015) Society: 111 bn/year CNY (2015)	Healthcare: 12 bn/year Society: 35 bn/year	Increased treatment costs, 3x multiplier for societal costs

*bn* billion, *CNY* Chinese Yen, *ECDC* European Centre for Disease Prevention and Control, *EMEA* European Medicines Agency, *G3CREC* third-generation cephalosporin-resistant *E. coli*, *MRSA* methicillin-resistant *Staphylococcus aureus*, *TB* tuberculosis, *tn* trillion, *USD* US Dollar

A separate approach has been to rely on national [48, 52, 53, 55] or global [19, 20] surveillance databases rather than individual sites. This offers greater external validity but may only be accurate in countries with representative patient data on healthcare utilisation, prescribing and antibiotic susceptibility, ideally linked to account for dependencies in these variables. The validity of global antibiotic use databases has been questioned, particularly in resource-poor settings, given the difficulty of capturing antibiotic supply from informal providers [58, 59]. Furthermore, cultures are less frequently taken in resource-poor settings and often only when patients do not seem to respond to empirical antibiotic therapy, leading to inflated estimates of ABR prevalence [59]. A better approach may be to estimate ABR costs using large prospective population-based or multi-centre studies where antibiotic susceptibility is tested in all patients seeking healthcare for acute infections.

When patient-level data are not available, studies have related ABR with costs on an ecological level (by subnational region [60, 61] or country [35]). The usual approach is to linearly regress the proportion of isolates that are resistant with healthcare expenditure (alongside other explanatory variables) [35, 60]. In some cases, an intermediate outcome, such as overall pathogen prevalence [61], is the primary outcome variable, which is then converted into costs.

### The impact of interventions

While most studies have focused on the overall ABR economic burden, some have examined the impact and cost-effectiveness of interventions that could potentially affect ABR. Most of these consider ABR alone as an exogeneous factor affecting the cost-effectiveness of an intervention but not itself influenced by the intervention. For instance, many cost-effectiveness evaluations of antibiotics have incorporated the effect of ABR on the effectiveness of antibiotic treatment [62] or the types of antibiotics that can be used [63]. Similarly, cost-effectiveness studies of interventions to reduce antibiotic consumption (such as stewardship programmes and rapid diagnostic tests) have typically considered intervention impact on antibiotic volume and costs. However, few studies have considered the potential impact of antibiotic consumption on (1) reducing ABR by preventing onward transmission of a bacteria susceptible to the antibiotic (positive externality) and/or (2) inducing ABR by increasing selective pressure (negative externality).

Some studies have captured these externalities using descriptive statistical approaches such as (1) using empirical data to evaluate the impact of a discrete intervention affecting antibiotic consumption (such as introduction of a stewardship programme) on ABR prevalence or treatment cost [64]; (2) estimating a fixed ratio between antibiotic consumption and ABR costs (or an intermediate proxy such as ABR prevalence or hospital bed-days used to treat infection with a particular pathogen) [54]; or (3) fitting a statistical model, such as linear correlation between antibiotic consumption and ABR costs (or an intermediate proxy), using data sampled over several countries and/or years [52].

These methods require an estimate of ABR costs. In hospital studies, this can be estimated as the cost of treating resistant infections in the hospital [64]. In ecological studies, ABR costs are often regarded as the cost of all antibiotics consumed in a single country and year [50, 65]. The most sophisticated methods derive from work by Phelps [37], who explicitly modelled market dynamics between antibiotic producers and consumers as well as the relationship between antibiotic consumption and the emergence of ABR. This was then used to estimate the total negative externality of annual antibiotic use in the USA, defined as the welfare loss due to ABR minus the welfare benefits that come from using antibiotics. This model was independently extended by Elbasha [47] and Kaier [66–68].

All the above methods make major simplifying assumptions, as described below:

- Some models assume that resistance against a particular antibiotic is only affected by the use of antibiotics of the same class. In practice, antibiotic use can affect ABR against an antibiotic of a different class. For instance, amoxicillin, which is typically prescribed for respiratory tract infections, is associated with increased trimethoprim and ciprofloxacin resistance in *Escherichia coli* causing urinary tract infections [69, 70]. Conversely, higher nitrofurantoin use was found to be associated with lower levels of trimethoprim and amoxicillin resistance, potentially due to collateral sensitivity or a negative correlation between resistance genes [69].
- Models relating (human) antibiotic consumption to ABR assume that the relationship is not confounded by between-country differences such as in infection prevention and control measures and agricultural antibiotics use. Reverse causality could play a role in cross-sectional data since physicians may avoid a particular antibiotic if ABR to that antibiotic is known to be high in that population [71]. In some cases, the potential role of reverse causality could be assessed using structural equation models or instrumental variables.
- Some models assume that the relationship is instantaneous, i.e. that a given level of antibiotic use will immediately result in some level of ABR. In practice, bacteria take time to acquire resistant genes and reach a new equilibrium prevalence in a population. Indeed, high levels of ABR may be the cumulative effect of years of antibiotic use, i.e. present antibiotic use may be depleting the health and wellbeing of future generations [72]. Furthermore, future changes in ABR can be unpredictable. While the emergence of mutations conferring ABR to certain antibiotics is predictable to some extent, the timing and impact of the introduction of new ABR genes into mobile genetic elements or widespread bacterial strains is not [10, 73].
- Models often assume that the relationship between antibiotic use and ABR is linear (or can be described with a simple function); this has some basis in ecological observations at the national level [74]. However, investigators using non-linear models suggest that the relationship is more complex and dynamic [3, 75, 76].

Advanced modelling approaches may circumvent some but not all of these limitations. Time-series approaches to modelling the relationship between population antibiotic use and ABR in hospitals suggest that this relationship is indeed non-linear [77]. Furthermore, machine learning approaches, such as boosted regression trees, can also be used, allowing flexibility in the functional relationship [70].

Dynamic effects, such as time delays and feedback loops, can be captured with transmission dynamic models [45, 78], dynamic Bayesian Markov models [79] or by including lagged covariates into regression analysis. Transmission dynamic models capture the effect of interventions on onward transmission and competition between susceptible and resistant bacterial strains, and can be used to track changes in ABR prevalence over time. Transmission dynamic models with strain competition that incorporate economic outcomes are rare [45, 78, 80]; one reason is that such models require data on antibiotic use in different settings, acquisition and carriage (duration) of antibiotic resistant bacteria, environmental swabs, movement of patients and contact patterns, rates of infection, and the associated current and future impact on patient outcomes and costs in order to predict the impact of interventions on ABR and the associated costs. However, some of them note that predictions about optimal policy may differ when economic considerations are incorporated into purely epidemiological/ecological models [3].

A key issue around all models is the reversibility of ABR, i.e. whether susceptible strains will eventually outcompete resistant strains when reducing selective pressure from antibiotic use. Most ABR mechanisms come with fitness costs that reduce the competitiveness of resistant strains compared to susceptible strains in the absence of antibiotic exposure [68]. However, reductions in antibiotic prescribing have not always led to reductions in ABR prevalence, potentially due to a lack of fitness costs, compensatory mutations that reduce fitness costs, and co-selection of ABR genes by other antibiotics. The reversibility of ABR likely depends on the setting, the bacterial species and on whether overall antibiotic use is reduced or one antibiotic is simply replaced by another [81]. Models that assume that ABR is not reversible effectively model antibiotic effectiveness as a non-renewable resource [3, 78]; these models aim to find strategies that obtain the greatest value from antibiotics before their effectiveness in exhausted.

## Discussion

### Current evidence base

While the number of studies that estimate the cost of ABR is rapidly accumulating, the majority of published studies still ignore several biases and have too narrow a focus to estimate the true cost of ABR. For example, we found no studies that adjusted for time-dependent confounding using an appropriate methodology when estimating the costs attributable to hospital-onset resistant infections nor any studies that examined the impact that future levels of ABR may have on clinical pathways. Completely ignoring time-dependent confounding likely leads to overestimation of the impact of ABR in the hospital setting given that patients that develop an ABR infection have likely deteriorated further between admission and acquisition than patients who remain infection free [23]. Attempting to correct for time-varying confounding using inappropriate methodology, such as standard regression techniques or multi-state models, may only partly remove the indirect effects of early infection that are mediated through the considered confounders, and may also introduce collider-stratification bias, which can lead to either an over- or underestimation of the true effect [14, 23, 26].

We also found few studies capturing wider costs beyond hospital treatment costs (such as productivity costs, out-of-pocket expenses, opportunity costs of lost bed days and inability to use antibiotics, and costs associated with the value of avoiding suffering), and few studies in low- and middle-income settings. Consequently, it is likely that most studies have overestimated current ABR costs in the hospital setting (because of incomplete control for biases and confounding) but underestimated total ABR costs (because of failure to account for wider societal costs and future consequences of ABR).

Lastly, we found a variety of approaches to costing, in line with previous reviews that concluded that heterogeneities in the quality of applied methods in ABR cost studies prevented meaningful comparisons [11, 14, 16–18].

### Conceptual framework

To address the limitations of existing studies, we have developed a conceptual framework to highlight the ideal scope and approaches for ABR cost estimations (Fig. 2). We recognise that an analysis that is robust and comprehensive according to this framework likely cannot be conducted at this time due to limitations in both data and analytical methods. Hence, our framework points to the need for better primary studies and surveillance to inform the development of more methodologically robust models of ABR costs. In Table 2, we outline some recommendations for the field.

**Table 2:** List of recommendations for future studies estimating the cost of antibiotic resistance (ABR) and related interventions

**Recommendations for primary data collection**	
• Capture all economic costs related to ABR in hospital patients, not just the directly observed outcomes such as increased length of stay	
• Explore use of g-methods to correct for both time-dependent biases and time-dependent confounders in studies evaluating time-varying exposures in hospital-based studies	
• Exhaustively investigate potential confounders that need to be collected and investigated in ABR cost studies, ideally using formal causal inference methods such as causal diagrams	
• Collect data on lost earnings and out-of-pocket expenses of patients and caregivers so that the wider household and societal costs of prolonged hospital stay and premature mortality can be captured; this is especially important in settings with high out-of-pocket medical expenses	
• Consider reporting measures of the psychosocial burden of suffering to patients and caregivers associated with illness, either by monetising the value of avoided suffering or by reporting this separately in units such as quality- or disability-adjusted life years	
• Consider both quality (internal validity) and broader representativeness (external validity) of data collected before extrapolating from study sites to wider regions such as the national or international level; if possible, data from multiple sites should be synthesised using meta-analysis or meta-regression (including geospatial variables, if appropriate)	
• Implications of ABR outside the hospital setting should be considered unless they are known to be negligible	
**Recommendations for further methodological development**	
• Investigate how levels of ABR may lead to increased costs for everyone, including patients with susceptible infections, those receiving antibiotics prophylactically and patients who are unable to access hospital beds because they are occupied by patients whose hospital stay has been extended by having a resistant infection	
• Explore the use of longitudinal data from prospective cohorts or large linked patient databases to understand the relationships between antibiotic use, ABR and costs of ABR	
• Ecological methods, such as regression, may allow extrapolation of site- or region-specific costs to a national or global level, adjusting for levels of ABR as well as other variables; however, further research is needed to investigate the implications of model simplifications, such as assuming linear and static relationships between antibiotic use and ABR, and the use of alternative modelling methods	
• Insights from transmission dynamic models of bacterial ecology and from economic models of antibiotic market dynamics need to be combined in order to inform optimal policies	
• Explore ways that long-term projections and macro-economic modelling can be incorporated into economic evaluations of ABR-related interventions	

### Limitations

The framework was informed by a narrative review of approaches and limitations that studies have taken to costing ABR. A narrative review aims to interpret and critique a large body of literature on a broad question, rather than summarise or synthesise literature on a narrowly focused topic [82]. In this case, it was appropriate because the question we are addressing is wide ranging, approaches to it are still being developed and the literature we reviewed is varied, including commentaries, primary data collection studies, economic evaluations and conceptual models. The narrative review was informed by a rapid literature search to obtain a broad overview of the methods used by relevant papers, combined with a review of references from previous reviews. The rapid methodological review found 110 relevant studies, far more than any other review of the topic in the literature. Nevertheless, the review methodology we used was not as exhaustive or unbiased as a full systematic review. In particular, the search terms and range of databases we searched were relatively limited because of the large number of relevant articles.

Our conceptual framework builds on gaps identified in current ABR estimates as well as on issues that have been previously discussed or that were considered important based on our own experience. Therefore, it is not an exhaustive list of all issues that could be encountered when estimating the costs of ABR.

## Conclusion

There is a clear need for more accurate estimates of the current and future costs attributable to ABR to inform decision-making around justified levels of investment in interventions that address the challenge of increasing ABR. Substantial improvements in methodological rigour and extension in time, perspective, scope and space are needed to capture the true costs of ABR in future studies. Furthermore, because better models cannot overcome data limitations, investment in prospective data collection is needed, including measurement of all relevant potential (time-varying) confounders as well as data on ABR infections and their associated costs and health consequences. This is particularly true in low-income settings where there is currently a clear lack of reliable data. Such studies need careful a priori consideration of potential confounders and biases. While this will be no easy task, strengthening the robustness of evidence on the true costs of ABR is critical to guide local, national and global efforts to address the issue.

## Supplementary information

**Additional file 1.** Search terms used in the rapid review.

**Additional file 2.** Data extracted from studies in the rapid review.

**Additional file 3.** Reporting items checklist based on Table 7.1 in Trico et al. Rapid Reviews to Strengthen Health Policy and Systems: A Practical Guide. Geneva: World Health Organisation; 2017.

## Data Availability

Data extracted from the rapid methodological review are included in this published article and in Additional file 2.
