# Supplementary material for: Quantifying the economic cost of antibiotic resistance and the impact of related interventions: rapid methodological review, conceptual framework and recommendations for future studies
Source: BMC Med. 2020 Mar 6;18:38. doi: 10.1186/s12916-020-1507-2 (PMC7059710; doi:10.1186/s12916-020-1507-2)
Supplement: Supplementary file 1 — Additional file 1. Search terms used in the rapid review. [file 12916_2020_1507_MOESM1_ESM.docx]

**Appendix 1. Search terms used in the rapid review.**

We conducted a rapid review of literature by searching PubMed and Ovid MEDLINE. We conducted two sets of searches: (i) a less sensitive search up to 17 Dec 2017 in selected fields only, and (ii) a more sensitive search from 18 Dec 2017 for articles published since the last search, without field restriction to ensure that we had better sensitivity for more recent articles.

Search terms used are listed below:

***PubMed***

(“cost” or “economics”) and (“antibiotic resistance” or “antimicrobial resistance” or “drug resistance”) in keywords and titles to 17 Dec 2017

(“cost” or “economics”) and (“antibiotic resistance” or “antimicrobial resistance” or “drug resistance”) in all fields from 18 Dec 2017 to 4 Nov 2019

***Ovid MEDLINE***

(“cost” or “economics”) and (“antibiotic resistance” or “antimicrobial resistance” or “drug resistance”) in abstracts, keywords and titles to 17 Dec 2017

(“cost” or “economics”) and (“antibiotic resistance” or “antimicrobial resistance” or “drug resistance”) in all fields from 18 Dec 2017 to 4 Nov 2019
