# Supplementary material for: Quantifying the economic cost of antibiotic resistance and the impact of related interventions: rapid methodological review, conceptual framework and recommendations for future studies
Source: BMC Med. 2020 Mar 6;18:38. doi: 10.1186/s12916-020-1507-2 (PMC7059710; doi:10.1186/s12916-020-1507-2)
Supplement: Supplementary file 3 — Additional file 3. Reporting items checklist based on Table 7.1 in Trico et al. Rapid Reviews to Strengthen Health Policy and Systems: A Practical Guide. Geneva: World Health Organisation; 2017. [file 12916_2020_1507_MOESM3_ESM.docx]

**Appendix 3. Reporting items checklist based on Table 7.1 in Trico et al. Rapid reviews to strengthen health policy and systems: a practical guide. Geneva: World Health Organization; 2017.**

| **Category** | **Items to consider** | **Answer** |
| --- | --- | --- |
| Protocol | Was a protocol used? If so, was the protocol made public, published in a journal, and/or registered (if so, provide reference and/or registration number, or link to protocol)? | Yes, a brief protocol was used as summarised in the Methods section. This was not published or registered. |
| Overall scope | Was the scope limited in any way? Were there a limited number of research or policy questions? Were the research questions of limited type (e.g. effectiveness only, specific populations)? Was the number of included studies limited? | Scope included all papers that quantified the cost of ABR from any economic perspective. There were no limitations on research questions or number of studies. |
| Comprehensiveness | Was the search strategy limited in any way (e.g. number of databases, grey literature, date, setting, language)? Were there limits on the types of study designs included (e.g. existing systematic reviews, randomized controlled trials)? Was textual analysis limited (e.g. no full-text review and/or limits on the number of items extracted)? | Search strategy was limited to simple keyword searches in PubMed and Ovid MEDLINE prior to 17 Dec 2017, and a more sensitive search to 4 Nov 2019. All study designs meeting inclusion and exclusion criteria were included. Full-text review of all shortlisted articles was performed. |
| Rigour and quality control | Was the process of dual study selection or dual data extraction modified or omitted? Was the internal or external review of the final research report limited or omitted? | No dual study selection was performed. Results were reviewed by all authors of this manuscript. |
| Synthesis | Was the assessment of risk of bias or quality of evidence limited or omitted? Was qualitative or quantitative analysis limited or omitted? | Simple descriptive statistical analyses were performed on key study characteristics.  Qualitative assessment was performed to extract key themes around study quality, limitation and biases.  Detailed analysis of individual papers was not conducted as the purpose of the review was to inform a methodological framework rather than to synthesise the results of the literature. |
| Other | When making statements about the findings of the rapid review, were the conclusions simplified or omitted? Is it appropriate to provide a disclaimer and/or limitations section in context with your findings? | The Discussion mentions that the review methodology was not sensitive enough to be fully comprehensive of the literature.  The conclusions were based on deductive reasoning on points that can be mostly referenced to previous reviews and commentaries, rather than requiring recourse to a comprehensive analysis of the literature. |
